# M. Pasteur Et La Rage

**Published:** 1887-09

**Authors:** 


**M. Pasteur et la Rage.** Par DR. LUTAND, Redacteur en Chef du *Journal de Medicine de Paris.* P. 440; 12mo.

That a prophet is not without honor, save in his own country, is a truth which certainly applies to M Pasteur. Dr. Lutand has been one of the most active and brilliant opponents of the Pasteurian system of preventive inoculations, and has been indefatigable in the collection of statistics to prove that as a prophylactic measure it has been an utter failure. Lutand takes as a motto one of Pasteur's own sayings: "The greatest fallacy of the human mind is to believe things to be true because we wish that they may be true." The author claims that Magendie discovered that dogs under certain conditions would not acquire rabies, but Galtier first studied the transmission of rabies from the dog to the rabbit, and that Dubois demonstrated that the nervous centres are the principal seat of the morbific virus. He even doubts the existence of any such disease as rabies in man, claiming with a considerable show of truth that the real disease is tetanus. Finally he claims that although rabies or tetanus has been produced in man in a number of cases by M. Pasteur, there is no evidence that it has been prevented or cured in a single case. On the whole, the book is very readable, and we recommend it to readers who desire exact information concerning the method and its results

